# The Use of Facebook in Recruiting Participants for Health Research Purposes: A Systematic Review

**DOI:** 10.2196/jmir.7071

**Published:** 2017-08-28

**Authors:** Christopher Whitaker, Sharon Stevelink, Nicola Fear

**Affiliations:** ^1^ Kings Centre for Military Health Research Department of Psychological Medicine Kings College London London United Kingdom

**Keywords:** epidemiology, social media, review, research

## Abstract

**Background:**

Social media is a popular online tool that allows users to communicate and exchange information. It allows digital content such as pictures, videos and websites to be shared, discussed, republished and endorsed by its users, their friends and businesses. Adverts can be posted and promoted to specific target audiences by demographics such as region, age or gender. Recruiting for health research is complex with strict requirement criteria imposed on the participants. Traditional research recruitment relies on flyers, newspaper adverts, radio and television broadcasts, letters, emails, website listings, and word of mouth. These methods are potentially poor at recruiting hard to reach demographics, can be slow and expensive. Recruitment via social media, in particular Facebook, may be faster and cheaper.

**Objective:**

The aim of this study was to systematically review the literature regarding the current use and success of Facebook to recruit participants for health research purposes.

**Methods:**

A literature review was completed in March 2017 in the English language using MEDLINE, EMBASE, Web of Science, PubMed, PsycInfo, Google Scholar, and a hand search of article references. Papers from the past 12 years were included and number of participants, recruitment period, number of impressions, cost per click or participant, and conversion rate extracted.

**Results:**

A total of 35 studies were identified from the United States (n=22), Australia (n=9), Canada (n=2), Japan (n=1), and Germany (n=1) and appraised using the *Critical Appraisal Skills Programme* (CASP) checklist. All focused on the feasibility of recruitment via Facebook, with some (n=10) also testing interventions, such as smoking cessation and depression reduction. Most recruited young age groups (16-24 years), with the remaining targeting specific demographics, for example, military veterans. Information from the 35 studies was analyzed with median values being 264 recruited participants, a 3-month recruitment period, 3.3 million impressions, cost per click of US $0.51, conversion rate of 4% (range 0.06-29.50), eligibility of 61% (range 17-100), and cost per participant of US $14.41. The studies showed success in penetrating hard to reach populations, finding the results representative of their control or comparison demographic, except for an over representation of young white women.

**Conclusions:**

There is growing evidence to suggest that Facebook is a useful recruitment tool and its use, therefore, should be considered when implementing future health research. When compared with traditional recruitment methods (print, radio, television, and email), benefits include reduced costs, shorter recruitment periods, better representation, and improved participant selection in young and hard to reach demographics. It however, remains limited by Internet access and the over representation of young white women. Future studies should recruit across all ages and explore recruitment via other forms of social media.

## Introduction

Social media is a popular Web-based tool that allows users to communicate and exchange information [1]. It allows digital content such as pictures, videos, and websites to be shared, discussed, republished, and endorsed by its users, their friends, and businesses. Adverts can be posted and promoted to specific target audiences by demographics such as region, age, or gender.

Social media has grown tremendously with Facebook, increasing from 6m to 1bn daily users from 2005 to 2015 [2]. This visibility lead to most social media sites monetizing adverts, with 92% of the private sector currently using social media as one of their employee recruitment strategies [3]. In 2014, 66% of the UK population used social media, with 96% of those users choosing Facebook [4]. It continued to grow in 2016, with 72% of the population using social media and 97% of them choosing Facebook [1].

Recruiting for health research is complex with strict requirement criteria imposed on the participants. Traditional research recruitment relies on flyers, newspaper adverts, radio and television broadcasts, letters, emails, website listings, and word of mouth. These methods are potentially poor at recruiting hard to reach demographics, can be slow, and expensive [5,6]. Recruitment via social media, in particular Facebook, may be faster and cheaper.

This paper aims to summarize the available evidence regarding Facebook as a recruitment tool for health research in terms of cost, speed, and its ability to find and represent hard to reach demographic groups (see Table 1 for common definitions). It will be compared with traditional methods and deemed successful if it shows equal or better costing and representation of target demographics. This will be the first systematic review the authors are aware of to summarize and appraise this data.

**Table 1:** Common definitions.

Impressions	The number of times that the ad is fetched (starts downloading to a computer or device)
Cost per click	The cost of advertising divided by the number of times the advert is clicked shown in USD ($)
Conversion rate	The number of people who click on the ad and then proceed to become paying customers, or in the case of research, participants (considered before their eligibility)
Eligibility	The percentage of participants who respond and are eligible for the trial. This reflects the specificity of ad campaigns targeting
Cost per participant	The cost of advertising divided by the eligible recruited participants

## Methods

A search of six databases, namely MEDLINE, EMBASE, Web of Science, PubMed, PsycInfo, Google Scholar, and an additional hand search of reference lists was performed in March 2017. It spanned the past 12 years due to the rise of social media from a negligible entity in 2006. A combination of the following keywords was implemented as a search strategy looking within the title or abstract:

Facebook, social media*, social network* AND internet, online, web* AND recruit*, research*, volunteer*, participant*, respondent*, patient select*, stud*, epidemiology, clinical*, health communication*, survey*

All the papers identified were exported to RefWorks [7], and duplicates were removed. Subsequently, the following exclusion criteria were applied: (1) Non-English language; (2) those not using Facebook as the recruitment tool; (3) those not recruiting for health research purposes; (4) those not constituting original research; (5) conference proceedings, letters to editors, posters, comments, and dissertations (due to difficulty accessing the full text and probable lack of detail); and (6) systematic reviews (although their reference lists were examined for eligible papers).

Full papers were appraised using the *Critical Appraisal Skills Programme* (CASP) checklist [8], and those deemed invalid were excluded (scoring less than 7/9; see Multimedia Appendix 1). Results tabulated included target demographic, number recruited, recruitment length, impressions, cost per ad click, conversion rate, eligibility, and cost per participant. Data was exported to Microsoft Excel for statistical analysis. Major outliers were removed (outside three standard deviations [SDs]), after which mean, median, and interquartile range were calculated.

## Results

### Summary of Accepted Studies

**Figure 1:**
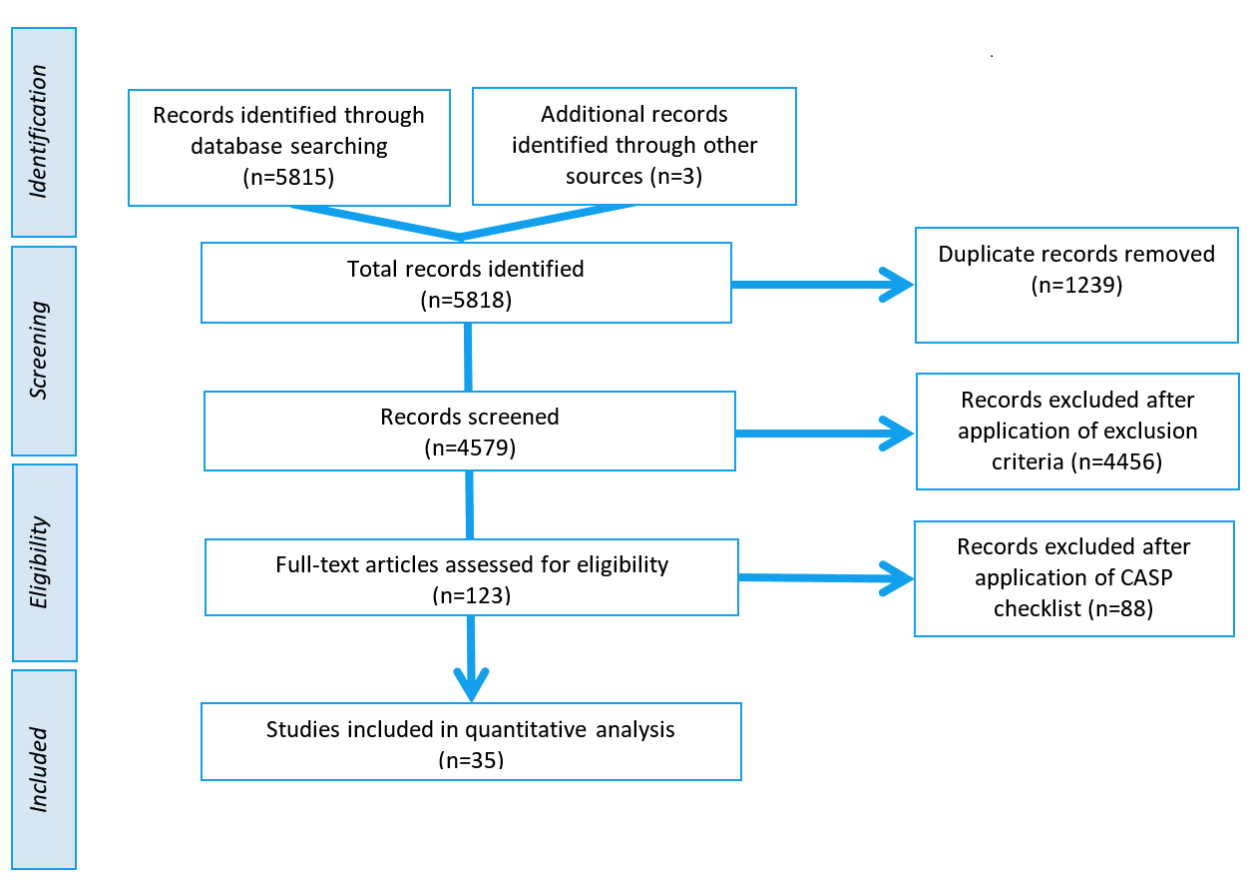
Article selection diagram.

A total of 5818 records were identified during the initial searches. Duplicates were removed (n=1239) and 4579 records were screened against the exclusion criteria (Figure 1). Additionally, 123 full papers were assessed for quality using the study design specific CASP checklist revealing 35 papers (scoring 7-9/9) to be included in the review (see Multimedia Appendix 1). Quantitative and qualitative data was tabulated (Tables 2 and 3), allowing comparison of cost and demographic recruited.

Most studies were conducted in the United States (n=22) with some in Australia (n=9) and Canada (n=2) and one in Japan and Germany, respectively. Some studies also tested interventions (n=10); three recruited for smoking cessation [6,9,10], two for *human papillomavirus* (HPV) vaccination [11,12], two for healthier lifestyle intervention [13,14]; and one each for perinatal studies [15], human immunodeficiency virus (HIV) prevention via soap opera viewing [3], and depression intervention [16]. Ten papers recruited those aged 18 years and over, 7 the age group of 18-25 years, and 16 recruited different ages (See Tables 3 and 4 for more demographic information).

**Table 2:** Extracted quantitative data from the 35 included papers.

Author	Number recruited	Recruitment length (months)^a^	Impressions (millions)	Cost per ad click (US $)^b^	Conversion rate (%), n numbers included where available	Eligibility (%), n numbers included where available	Cost per participant (US $)^b^
Adam LM (2016) [15]	45	0.8	0.04	0.21^b^	NR^c^	56 (n=45)	15.12^b^
Admon L (2016) [29]	1178	1.0	0.36	0.58	13.2 (n=1592)	74 (n=1178)	14.63
Akard TF (2015) [17]	106	2.0	3.90	1.08	3.0	61 (n=106)	17
Arcia, A (2014) [18]	344	4.0	10.50	0.63	6.0	50	11.11
Batterham PJ (2014) [28] stage 1	610	0.1	NR	NR	3.0	NR	9.82^b^
Batterham PJ (2014) [28] stage 2	1283	0.1	NR	NR	3.0	NR	1.51^b^
Bauermeister JA (2012) [30]	22	NR	NR	NR	NR	NR	NR
Bull S (2013) [31]	1578	36.0^d^	NR	NR	NR	NR	NR
Carlini B (2015) [19]	285	4.0	NR	NR	NR	NR	8.92
Carter-Harris L (2016) [9]	331	0.6	0.06	0.45	29.5	NR	1.51
Child RJH (2014) [20]	78	0.1	NR	NR	NR	NR	NR
Chu JL (2013) [21]	88	9.0	17.50	0.39^b^	5.0 (n=180)	49	15.35^b^
Close S (2013) [22]	39	0.2	2.50	0.61	18.0	NR	19.44
Crosier BS (2016) [32]	264	1.0	0.01	0.20	NR	NR	8.14
Fenner Y (2012) [33]	278	4.0	36.10	0.48^b^	4.0	NR	14.50^b^
Frandsen TL (2014) [6]	138	19.0	14.50	0.68^b^	NR	NR	30.48^b^
Frandsen M (2016) [10]	92	13.5	NR	NR	NR	61	74.64^b^
Harris ML (2015) [34]	NR	8.0	NR	0.51^b^	2.0	93 (n=3795)	8.55^b^
Jones R (2015) [35]	230	1.0	NR	0.36	3.0	39	37.74
Kappa JM (2013) [36]	0	0.3	0.90	0.98	3.0	78 (n=280)	NR
Miyagi E (2014) [37]	126	9.0	5.70	NR	NR	95	NR
Moreno MA (2017) [14]	8	NR	NR	NR	NR	NR	40.99
Morgan AJ (2015) [16]	35	11.0	2.00	0.45^b^	NR	NR	14.32^b^
Musiat P (2016) [38]	26	3.0	0.50	1.74^b,d^	0.1	90	76.15^b^
Nelson EJ (2014) [24]	1003	2.0	NR	NR	48.0^d^(n=1003)	91	1.36
Parkinson S (2013) [39]	100	0.2	1.30	NR	15.0	83	NR
Pedersen ER (2014) [25]	1023	1.0	3.30	0.38	5.0	45	7.05
Ramo DE (2014) [40]	1548	13.0	28.70	0.45	1.0	NR	4.28
Ramo DE (2012) [41]	230	2.0	3.20	0.34	9.0	51	8.80
Raviotta JM (2016) [11]	428	6.0	21.00	1.24	NR	NR	110.00^d^
Remschmidt C (2014) [42]	1161	2.0	62.90	NR	9.0	NR	NR
Schumacher KR (2014) [26]	394	12.0	NR	NR	NR	100 (n=394)	NR
Schwinn T (2017) [43]	797	4.2	187.00^d^	0.6	2.8	43 (n=1873)	51.70
Staffileno BA (2016) [13]	23	18.0	NR	0.73	NR	17	NR
Subasinghe AK (2016) [12]	919	13.0	55.40	0.67^b^	NR	NR	17.29^b^
Yuan P (2014) [27]	1404	4.0	NR	NR	NR	NR	3.56

^a^Calculated as a percentage of a 31-day month.

^b^AUD converted to USD with 0.72 and CD to USD with 0.75 exchange rates where appropriate.

^c^NR: not reported; not reported if data unavailable.

^d^Outliers of over 3 standard deviations excluded from statistical calculation.

**Table 3:** Extracted qualitative data (Authors G-Z) from the 35 included papers.

Author	Country	Target demographic^a^	Comparison to control demographic
Harris ML (2015) [34]	Australia	18-23 years	Partly representative; higher proportion of female and tertiary education
Jones R (2015) [23]	United States	18-29 years, female, in a sexual relationship with at least one man in past 3 months	Partly representative; higher proportion of education
Kappa JM (2013) [36]	United States	35-49 years, female	No comparison made
Miyagi E (2014) [37]	Japan	16-35 years, female	Partly representative; higher proportion of 26-35 age group and a low BMI^b^, and lower proportion of 16-21 age group
Moreno MA (2017) [14]	United States	14-18 years	No comparison made
Morgan AJ (2015) [16]	Australia	No other criteria	No comparison made
Musiat P (2016) [38]	Australia	18-25 years	No comparison made
Nelson EJ (2014) [24]	United States	18-30 years, lives in metropolitan area	Partly representative; higher proportion of HPV^c^vaccination
Parkinson S (2013) [39]	Australia	18-25 years	Partly representative; higher proportion of females, university education, unemployed and high income rate, and lower proportion of full time employment
Pedersen ER (2014) [25]	United States	18-34 years, previously served in the US Air Force, Army, Marine Corps, Navy	Partly representative; higher proportion of Hispanic or Latino and lower proportion of black or African American
Ramo DE (2014) [40]	United States	18-25 years, smoker	Partly representative; higher proportion of white and males
Ramo DE (2012) [41]	United States	18-25 years	Partly representative; higher proportion of white and males
Raviotta JM (2016) [11]	United States	18-25, male, student, lives in Pittsburgh	Partly representative; higher proportion of homo or bisexual and social media use
Remschmidt C (2014) [42]	Germany	18-25 years	Representative
Schumacher KR (2014) [26]	United States	15-18 years, parents of <15 years, Fontan-associated protein losing enteropathy, plastic bronchitis	Representative
Schwinn T (2017) [43]	United States	13-14 years, female	Partly representative; higher proportion of African American and less reported parents completing high school. Smoking, drinking, and drugs use was representative
Staffileno BA (2016) [13]	United States	18-45 years, prehypertension	No comparison made
Subasinghe AK (2016) [12]	Australia	18-25 years, in Victoria who had not been vaccinated against HPV	Representative
Yuan P (2014) [27]	United States	HIV^d^positive	No comparison made

^a^Assume all are over 18 years and English speaking unless otherwise stated.

^b^BMI: body mass index.

^c^HPV: *human papillomavirus.*

^d^HIV: human immunodeficiency virus.

**Table 4:** Extracted qualitative data (authors A-F) from the 35 included papers.

Author	Country	Target demographic^a^	Comparison with control demographic
Adam LM (2016) [15]	Canada	23-40 years, female, <25 miles from center, 8-20 weeks pregnant	No comparison made
Admon L (2016) [29]	United States	African American or Hispanic interested in pregnancy	Partly representative; higher proportion of African Americans, high income, pregnancy, and reporting fair or poor health
Akard TF (2015) [17]	United States	Parents of children or teenagers	Partly representative; higher proportion of white and female
Arcia, A (2014) [18]	United States	18-44 years, nulliparous, >20 weeks gestation	Partly representative; higher proportion of younger age groups
Batterham PJ (2014) [28] stage 1	Australia	No other criteria	Partly representative; higher proportion of education, females, young adults, and lower levels of young adolescents
Batterham PJ (2014) [28] stage 2	Australia	No other criteria	No comparison made
Bauermeister JA (2012) [30]	United States	18-24 years	Partly representative; higher proportion of white ethnicity and tertiary education and lower proportion of cigarette use
Bull S (2013) [31]	United States	15-24 years	Representative
Carlini B (2015) [19]	United States	Brazilian and Portuguese speakers	No comparison made
Carter-Harris L (2016) [9]	United States	55-77 years, current or ex-smokers	No comparison made
Child RJH (2014) [20]	United States	Emergency nurses	Representative
Chu JL (2013) [21]	Canada	15-24 years, PTSD^b^	Partly representative; higher proportion of females and younger adults
Close S (2013) [22]	United States	Any age, Klinefelter syndrome	Representative
Crosier BS (2016) [32]	United States	Self-reports auditory hallucinations	Partly representative; higher proportion females
Fenner Y (2012) [33]	Australia	16-25 years, female	Partly representative; higher proportion of increased BMI^c^
Frandsen TL (2014) [6]	Australia	Smoking >10 cigarettes per day for 3+ years, not enrolled in a cessation trial in the last 3 months	Partly representative; higher proportion of young adults
Frandsen M (2016) [10]	Australia	Smokers 10+ per day, 3 years +, no intention to quit next month, >25km from city center	No comparison made

^a^Assume all are over 18 years and English speaking unless otherwise stated.

^b^PTSD post-traumatic stress disorder.

^c^BMI: body mass index.

Other than basic demographic information including age and sex, most papers recruited participants with specific characteristics (n=18), including parents of children [17], nulliparous women at the beginning of their pregnancy [18], Brazilian and Portuguese speakers [19], emergency nurses [20], those with post-traumatic stress disorder (PTSD) [21], those with Klinefilter syndrome [22], those in sexual relationships [23], those living in a metropolitan area [24], US veterans [25], parents of children with Fontan-associated protein losing enteropathy [26], and those of positive HIV status [27]. Two papers [16,28] had no targeting features except being over 18 years old.

### Summary of Quantitative Data

There were several pieces of data that outlay three SDs and so were removed from statistical analysis, namely, a recruitment length of 36 months [31], an impression count of 127 million [43], a cost per click of US $1.74 [38], a conversion rate of 48% [24], and a cost per participant of US $110.00 [11].

Table 5 shows median data: 264 recruited participants, a 3-month recruitment period, 3.3 million impressions, cost per click of US $0.51, conversion rate of 4% (range 0.06-29.50), eligibility of 61% (range 17-100), and cost per participant of US $14.41.

**Table 5:** Statistical analysis of extracted data with outliers removed.

Form of distribution analysis	Number recruited	Recruitment length (months)	Impressions (millions)	Cost per ad click (US $)	Conversion rate (%)	Eligibility (%)	Cost per participant (US $)
Mean	463	5.13	12.9	0.57	7	65	19.77
Median	264	3.00	3.3	0.51	4	61	14.41
Interquartile range	775	8.00	16.6	0.28	6	39	10.66

### Target Population

Most studies (n=24) compared their recruited participants with either a control group recruited by traditional methods or to national data. This showed the recruited participants to be relatively representative except for some minor differences:

- There was over representation of females in 5 papers [17,21,28,32,39] and of males in 2 papers [40,41].
- Four papers reported an over representation of white ethnicity [17,30,40,41], two of African American representation [29,43], and 1 an over representation of Hispanic or Latino ethnicities [25].
- Four papers suggested over representation of a young adult group [9,18,21,28], including Frandsen M (2014), who found Web-based age to be significant younger than from the control groups recruited by mail, newspaper ads, and flyers.
- Four papers reported a higher degree of education [23,30,34,39] and two a higher rate of income [29,39] than that of the general population.
- Fenner Y (2012) reported an over representation of people with a higher body mass index (BMI) in Australia [33], whereas Miyagi, E (2014) reported an over representation of low BMI in Japan [37].
- Nelson EJ (2014) reported a higher rate of HPV vaccination [24] than predicted in Australia, whereas Remschmidt C (2014) shows it to be representative of the general population in Germany [42].
- Bauermeister JA (2012) showed the participants to be representative of the general population for alcohol consumption, marijuana, ecstasy, and cocaine use [30], with Jones R (2012) showing representation of marijuana use, sexually transmitted infection (STI) rates, and sexual relationship history [35].
- Full time employment [25] and nonsmoker status [30] where each under represented once compared with the general population.

## Discussion

This paper summarizes the available evidence regarding the success of Facebook as a recruitment tool for research purposes. Some of the results can only be compared with Web-based advertising, namely, the impressions, cost per click, and conversion rate as traditional recruitment uses different markers. The remaining data on recruitment number, length of study, eligibility, and cost per participant can be compared widely with other forms of traditional recruitment.

### Facebook Compared With Web-Based Advertising

Cost per click only varied slightly across studies, especially when targeting similar groups. The median cost per click from this review was US $0.51 compared with US $0.27—the mean cost per click on Facebook as a whole [44]. This shows people are less likely to interact with a health recruitment ad. The conversion rate of 4% can also be compared with the mean value of 2.4% across all Web-based advertising [45]. This suggests that although people are less interested in health research ads overall, those who do interact with them are more likely to convert. This increase in conversion rate however, doesn’t appear large enough to counteract the increased cost per click with health recruitment still costing more than general advertising.

### Facebook Compared With Traditional Methods

The cost per participant on Facebook was shown to be less than traditional methods. Our median value of US $14.41 compares favorably with rates suggested by Tate, D (2014) of US $1094.27 per participant for television recruitment, US $811.99 for printed media, US $635.92 for radio, and US $37.77 for email when recruiting for a survey on English language competency [5]. Carlini BH (2015) had similar findings with a mean cost per participant of US $16.22 via Google ads, and between US $13.12 and US $250.00 for other traditional methods when recruiting young adults for weight gain analysis [19].

The cost per participant values contained a major outlier; Raviotta JM (2016) reported a cost of US $110 per recruited participant [11]. The cost per click of the study (US $1.24) fell slightly outside one SD of the median and did not explain the increased cost per participant. On closer inspection, the reason for the expense became clear, “the difference in time and effort required to complete a 7-13 month study with two blood draws and three vaccine injections vs. a 30 minute survey...explains the increased cost” [11].

### Facebook Compared With Other Social Media Sites

Three articles simultaneously used other social media sites to recruit participants, namely Twitter and MySpace. Bull S (2013) used MySpace but found it unsuccessful in recruiting any participants [31]. This is unsurprising considering the massive drop in MySpace primary users from 2008 to 2011 [46]. Harris, M. L (2015) implemented recruitment via Facebook and Twitter but changed to use Facebook alone due to its increased success [34]. Yuan P (2014) also used Twitter alongside Facebook. The study received 10,006 Facebook ad clicks and 161 Twitter interactions. It was found that the number of Facebook ad clicks was moderately correlated to the number recruited (*r*=.52 *P*<.001) but that there was little correlation between Twitter interactions and links clicked (*r*=.17, *P*=.06; *r*=.18, *P*=.06, respectively) [27]. These findings suggest Facebook is a superior recruitment tool when compared with Twitter and MySpace, although there is limited analysis across the three papers. This was an interesting albeit unintentional finding, but more research should be carried out in this area before making conclusions.

### Facebook’s Representation of the Population

Sociodemographic characteristics of the recruited participants were compared either with traditionally recruited participants or to available national statistics. Alcohol consumption; marijuana, ecstasy, and cocaine use [30]; STI rates; and sexual relationship history [35] were found to be representative of the total population. Those who use recreational drugs and have at risk sexual behavior tend to be found in hard to reach populations. The fact these studies mirror national statistics highlights the power of social media to target specific populations. Traditional methods tend to under represent these groups [47], meaning Facebook recruitment potentially yields more significant results.

Another point highlighting the success of Facebook recruitment would be the differing BMI results from Miyagi E (2014) and Fenner Y (2012), with the latter Australian paper showing a considerably higher average than the Japanese study. This simply shows the different obesity rates of the two countries. Australia reports 64% of its population to have a BMI above 25kg/m^2^compared with 24% in Japan [48]. This also seems true for the differences in reported rates of HPV vaccination between Nelson EJ (2014) and Remschmidt C (2014); 39.7% of adolescent females in America [49] are vaccinated compared with 49% of Germans [50].

Other demographic data was found to be representative of the target population and comparable with traditional recruitment with only a few exceptions:

There was an over representation of white ethnicity. Facebook claims to be diverse [51], but papers suggest either these claims are not true, targeted marketing misses certain ethnicities, or that different groups have different response rates. This over representation is also shown in a review of traditional methods by Yancey AK (2005), suggesting the problem is not limited to social media recruitment [52].

Four papers also showed over representation of females. This may be due to the fact that a higher percentage of women use Facebook [1] or because fewer men respond to recruitment in general [53]. Brown WJ (1998) found similar results using traditional methods, again suggesting the problem is not limited to Facebook [54].

Education and income are often confounding factors, and it is perhaps unsurprising to find over representation in both these areas. This is comparable again with traditional recruitment methods, with Gorelick PB (1998) finding that more years in education increased the likelihood of entering and completing a clinical trial with those of lower levels “not wanting to be guinea pigs” [55].

### Strengths and Limitations

Strengths of this review include the wide search ensuring all available literature was gathered and the detailed cost analysis. The main limitation is the relatively small number of studies available with numerical data on costings and population comparisons. Several papers had substantial recruitment numbers (n=1578 [30), but many were small (n=26 [13]), reducing the reliability. Most papers focused on ages in the range of 18-30 years. Carter-Harris L (2016) [9] recruited those aged 55-77 years, showing that although older people may be less likely to adopt newer technologies (of those over 65 years, 48% are active Facebook users compared with 64% for 50-64 year olds, 79% for 30-49 year olds, and 82% for 18-29 year olds [56]), recruitment can still be successful, reporting US $1.51 cost per participant. The expected barrier from lack of Internet access or experience in the older population is smaller than most think.

The percentage of people with access to the Internet is steadily increasing [1], and procedural methods can be put into place to prevent this misrepresentation of data [57]. Young, SD (2013) found that even 79% of homeless youths manage to access social media sites once per week [58]. Although Internet access currently remains to be a barrier, it seems to be smaller than barriers facing traditional methods and is set to improve in the future.

### Conclusions

There is growing evidence to suggest that Facebook is a successful recruitment tool, and its use, therefore, should be considered when implementing future health research. Benefits include reduced cost, shorter recruitment periods, better representation, and improved participant selection in young and hard to reach demographics. This may spell the end for traditional methods, although currently the minor limitations of Internet access and the over representation of young white women may make its use inappropriate in some settings.

## Appendix

Multimedia Appendix 1 Cohort study CASP checklist scores – score out of first 9 questions.

## Abbreviations

HIV: human immunodeficiency virus
HPV: human papillomavirus
PTSD: post-traumatic stress disorder
