# Supplementary material for: The Use of Facebook in Recruiting Participants for Health Research Purposes: A Systematic Review
Source: J Med Internet Res. 2017 Aug 28;19(8):e290. doi: 10.2196/jmir.7071 (PMC5594255; doi:10.2196/jmir.7071)
Supplement: Multimedia Appendix 1 [file jmir_v19i8e290_app1.pdf]

Multimedia Appendix 1.

Author	Score/9
Adam LM. (2016) [1]	8
Admon L (2016) [2]	9
Akard TF (2015) [3]	8
Arcia, A (2014) [4]	7
Batterham PJ (2014) [5]	7
Bauermeister JA (2012) [6]	7
Bull S (2013) [7]	7
Carlini B (2015) [8]	7
Carter-Harris L (2016) [9]	7
Child RJH (2014) [10]	7
Chu JL (2013) [11]	7
Close S (2013) [12]	7
Crosier BS (2016) [13]	8
Fenner Y (2012) [14]	7
Frandsen TL (2014) [15]	7
Frandsen M (2016) [16]	8
Harris M (2015) [17]	7
Jones R (2015) [18]	8
Kappa JM (2013) [19]	7
Miyagi E (2014) [20]	7
Moreno MA (2017) [21]	7
Morgan AJ (2015) [22]	8
Musiat P (2016) [23]	8
Nelson EJ (2014) [24]	8
Parkinson S (2013) [25]	7
Pedersen ER (2014) [26]	8
Ramo DE (2014) [27]	9
Ramo DE (2012) [28]	8
Raviotta JM (2016) [29]	8
Remschmidt C (2014) [30]	7
Schumacher KR (2014) [31]	7
Schwinn T (2017) [32]	7
Staffileno BA (2016) [33]	7
Subasinghe AK (2016) [34]	8
Yuan P (2014) [35]	7

Appendix References

1. Adam LM, Manca DP, Bell RC. Can Facebook Be Used for Research? Experiences Using Facebook to Recruit Pregnant Women for a Randomized Controlled Trial. 2016 J Med Internet Res, 18(9), 250. PMID:5052464
2. Admon L, Haefner JK, Kolenic GE, Chang T, Davis MM. Recruiting Pregnant Patients for Survey Research: A Head to Head Comparison of Social Media-Based Versus Clinic-Based Approaches. 2016 J Med Internet Res. 18(2), 326. PMID:5215244
3. Akard TF, Wray S, Glimmer MJ. Facebook advertisements recruit parents of children with cancer for an online survey of web-based research preferences. 2015 Cancer Nursinig 38(2), 155-161. PMID:24945264
4. Arcia A. Facebook advertisements for inexpensive participant recruitment among women in early pregnancy. 2014 Health Education & Behavior 41(3), 237-241. PMID:24082026
5. Batterham PJ. Recruitment of mental health survey participants using internet advertising: Content, characteristics and cost effectiveness. 2014 International Journal of Methods in Psychiatric Research 23(2), 184-191. PMID:24615785
6. Bauermeister JA, Zimmerman MA, Johns MM, Glowacki P, Stoddard S, Volz E. Innovative recruitment using online networks: Lessons learned from an online study of alcohol and other drug use utilizing a web-based, respondent-driven sampling. 2012 Journal of Studies on Alcohol and Drugs 73(5), 843-838. PMID:22846248
7. Bull SS. Recruitment and retention of youth for research using social media: Experiences from the Just/Us study. 2013 Vulnerable Children and Youth Studies 8(2), 171-181. DOI: 10.1080/17450128.2012.748238
8. Carlini BH, Safioti L, Rue TC, Miles L. Using internet to recruit immigrants with language and culture barriers for tobacco and alcohol use screening: A study among brazilians. 2015 Journal of Immigrant and Minority Health 17(2), 553-560. PMID:24563138
9. Carter-Harris L, Ellis RB, Warrick A, Rawl S. Beyond Traditional Newspaper Advertisement: Leveraging Facebook-Targeted Advertisement to Recruit Long-Term Smokers for Research. 2016 J Med Internet Res. 18(6), 117. PMID:4927805
10. Child RJ, Mentes JC, Pavlish C, Phillips LR. Using facebook and participant information clips to recruit emergency nurses for research. 2014 Nurse Researcher 21(6), 16-21. PMID:25059083
11. Chu JL, Snider C,. Use of a social networking web site for recruiting canadian youth for medical research. 2013 Journal of Adolescent Health 52(6), 792-794. PMID:23352727
12. Close S, Smaldone A, Fennoy I, Reame N, Grey M. Using information technology and social networking for recruitment of research participants: Experience from an exploratory study of pediatric klinefelter syndrome. 2013 Journal of Medical Internet Research 15(3), 171-181. PMID:23512442
13. Crosier S, Brian RM, Ben-Zeev D. Using Facebook to Reach People Who Experience Auditory Hallucinations. 2016 J Med Internet Res. 18(6), 160. PMID:4925933
14. Fenner Y, Garland SM, Moore EE, Jayasinghe Y, Fletcher A, Tabrisi SN, Gunasekaran B, Wark JD. Web-based recruiting for health research using a social networking site: An exploratory study. 2012 Journal of Medical Internet Research 14(1), 214-227. PMID:22297093
15. Frandsen M, Walters J, Ferguson SG. Exploring the viability of using online social media advertising as a recruitment method for smoking cessation clinical trials. 2014 Nicotine & Tobacco Research 16(2), 247-251. PMID:24127266
16. Frandsen M, Thow M, Ferguson SG. The Effectiveness Of Social Media (Facebook) Compared With More Traditional Advertising Methods for Recruiting Eligible Participants To Health Research Studies: A Randomized, Controlled Clinical Trial. 2016 JMIR Res Protoc. 5(3), 161. PMID:4997003

17. Harris ML, Loxton D, Wigginton B, Lucke JC. Recruiting online: Lessons from a longitudinal survey of contraception and pregnancy intentions of young australian women. 2015 American Journal of Epidemiology 181(10), 737-746. DOI:10.1093/aje/kwv006
18. Jones R, Lacroix LJ, Nolte K. “Is your man stepping out?" an online pilot study to evaluate acceptability of a guide-enhanced HIV prevention soap opera video series and feasibility of recruitment by facebook advertising. 2015 Journal of the Association of Nurses in AIDS Care. 26(4), 368-386. PMID:26066692
19. Kapp JM, Peters C, Olover DP. Research recruitment using facebook advertising: Big potential, big challenges. 2013 Journal of Cancer Education: The Official Journal of the American Association for Cancer Education 28(1), 134-137. PMID:23292877
20. Miyagi E, Motoki Y, Asai-Sato M, Taguri M, Morita S, Hirahara F, Wark JD, Garland SM. Web-based recruiting for a survey on knowledge and awareness of cervical cancer prevention among young women living in kanagawa prefecture, japan. 2014 International Journal of Gynecological Cancer 24(7), 1347-1355. PMID:25054449
21. Moreno MA, Waite A, Pumper M, Colburn T, Holm M, Mednoza J. Recruiting Adolescent Research Participants: In-Person Compared to Social Media Approaches. 2017 Cyberpsychol Behav Soc Netw, 20(1), 64-7. PMID:27976951
22. Morgan AJ, Jorm AF, Mackinnon AJ. Internet-based recruitment to a depression prevention intervention: Lessons from the mood memos study. 2013 Journal of Medical Internet Research 15(2), 31. PMID:23403043
23. Musiat P, Winsall M, Orlowski S, Antezana G, Schrader G, Battersby M, Bidargaddi N. Paid and Unpaid Online Recruitment for Health Interventions in Young Adults. 2016 Paid and Unpaid Online Recruitment for Health Interventions in Young Adults. 59(6), 662-7. PMID:27663927
24. Nelson EJ, Hughes J, Oakes M, Pankow JS, Kulasingam SL. Estimation of geographic variation in human papillomavirus vaccine uptake in men and women: An online survey using facebook recruitment. 2014 Journal of Medical Internet Research 16(9), 109-119. PMID:25231937
25. Parkinson S, Bromfield L. Recruiting young adults to child maltreatment research through facebook: A feasibility study. 2013 Child Abuse and Neglect 37(9), 716-720. PMID:23768931
26. Pedersen ER, Helmuth ED, Marshall GN, Schell TL, PunKay M, Kurz J. Using facebook to recruit young adult veterans: Online mental health research. 2015 JMIR Research Protocols 4(2), 63. PMID:26033209
27. Ramo DE, Rodrigues TM, Chavez K, Sommer MJ, Prochaska JJ. Facebook recruitment of young adult smokers for a cessation trial: Methods, metrics, and lessons learned. 2014 Internet Interventions 1(2), 58-64. PMID:25045624
28. Ramo DE, Prochaska JJ. Broad reach and targeted recruitment using facebook for an online survey of young adult substance use. 2012 Journal of Medical Internet Research 14(1), 228-237. PMID:22360969
29. Raviotta JM, Nowalk MP, Lin CJ, Huang H, Zimmerman RK. Using Facebook™ to recruit college-age men for a Human Papillomavirus vaccine trial. 2017 Am J Mens Health, 10(2), 110-9. PMID:4427547
30. Remschmidt C, Walter D, Schmich P, Wetzstein M, Delere Y, Wichmann O. Knowledge, attitude, and uptake related to human papillomavirus vaccination among young women in germany recruited via a social media site. 2014 Human Vaccines and Immunotherapeutics 10(9), 2527-2535. PMID:25483492
31. Schumacher KR, Stringer KA, Donohue JE, Yu S, Shaver A, Caruthers RL, Zikmund-Fisher BJ, Fifer C, Goldberg C, Russell MW. Social media methods for studying rare diseases. Pediatrics. 2014 Pediatrics 133(5), 1345-1353. PMID:24733869
32. Schwinn T, Hopkins J, Schinke S, Liu X. Using Facebook ads with traditional paper mailings to recruit adolescent girls for a clinical trial. 2017 Adictive Behaviors. 65, 207-13.
33. Staffileno BA, Zschunke J, Weber M, Gross LE, Fogg L, Tangney CC. The Feasibility of Using Facebook,

Craigslist, and Other Online Strategies to Recruit Young African American Women for a Web-Based Healthy Lifestyle Behavior Change Intervention. 2016 J Cardiovascular Nurs. 1(10), 10. PMID:27428356

34. Subasinghe A, Nguyen M, Wark JD, Tabrizi SN, Garland SM. Targeted Facebook Advertising is a Novel and Effective Method of Recruiting Participants into a Human Papillomavirus Vaccine Effectiveness Study. 2016 JMIR Res Protoc, 5(3), 154. PMID:4975794
35. Yuan P, Bare MG, Johnson OM, Saberi P. Using online social media for recruitment of human immunodeficiency virus-positive participants: A cross-sectional survey. 2014 Journal of Medical Internet Research 16(5), 101-109. PMID:24784982
